# Alterations in brain structure and function in patients with osteonecrosis of the femoral head: a multimodal MRI study

**DOI:** 10.7717/peerj.11759

**Published:** 2021-08-20

**Authors:** Jie Ma, Jia-Jia Wu, Xu-Yun Hua, Mou-Xiong Zheng, Bei-Bei Huo, Xiang-Xin Xing, Sheng-Yi Feng, Bo Li, Jianguang Xu

**Affiliations:** 1 School of Rehabilitation Science, Shanghai University of Traditional Chinese Medicine, Shanghai, China; 2 Center of Rehabilitation Medicine, Yueyang Hospital of Integrated Traditional Chinese and Western Medicine, Shanghai University of Traditional Chinese Medicine, Shanghai, China; 3 Yangzhi Rehabilitation Hospital (Shanghai Sunshine Rehabilitation Center), Tongji University, Shanghai, China; 4 Department of Traumatology and Orthopedics, Yueyang Hospital of Integrated Traditional Chinese and Western Medicine, Shanghai University of Traditional Chinese Medicine, Shanghai, China

**Keywords:** Functional magnetic resonance imaging, Diffusion tensor imaging, Pain, Functional plasticity, Osteonecrosis of the femoral head

## Abstract

**Background:**

Pain, a major symptom of osteonecrosis of the femoral head (ONFH), is a complex sensory and emotional experience that presents therapeutic challenges. Pain can cause neuroplastic changes at the cortical level, leading to central sensitization and difficulties with curative treatments; however, whether changes in structural and functional plasticity occur in patients with ONFH remains unclear.

**Methods:**

A total of 23 ONFH inpatients who did not undergo surgery (14 males, nine females; aged 55.61 ± 13.79 years) and 20 controls (12 males, eight females; aged 47.25 ± 19.35 years) were enrolled. Functional indices of the amplitude of low-frequency fluctuation (ALFF), regional homogeneity (ReHo), and a structural index of tract-based spatial statistics (TBSS) were calculated for each participant. The probability distribution of fiber direction was determined according to the ALFF results.

**Results:**

ONFH patients demonstrated increased ALFF in the bilateral dorsolateral superior frontal gyrus, right medial superior frontal gyrus, right middle frontal gyrus, and right supplementary motor area. In contrast, ONFH patients showed decreased ReHo in the left superior parietal gyrus and right inferior temporal gyrus. There were no significant differences in TBSS or probabilistic tractography.

**Conclusion:**

These results indicate cerebral pain processing in ONFH patients. It is advantageous to use functional magnetic resonance imaging to better understand pain pathogenesis and identify new therapeutic targets in ONFH patients.

## Introduction

Osteonecrosis of the femoral head (ONFH) is a challenging orthopedic disease associated with pain and joint dysfunction. ONFH is frequently observed in the clinic and severely reduces patient quality of life. In recent years, tens of thousands of new cases of osteonecrosis have been diagnosed in different countries (Hopkins & Genant, 2020; Hungerford, 2002). Pain is a major symptom of ONFH and is typically confined to the groin area; however, pain may occasionally occur in the ipsilateral hip, knee, or greater trochanteric area (Zhao & Hu, 2012). Furthermore, analgesics have demonstrated limited efficacy in ONFH treatment. Previous studies have found that pain is a complex sensory and emotional experience involving psychobiology, expectations of past and learned pain experiences, and attentional processes (Morton, Sandhu & Jones, 2016). Pain can induce functional neuroplastic changes at the cortical level, leading to central sensitization, which may, in turn, aggravate pain itself (May, 2008). Therefore, exploring the central plasticity of ONFH may help physicians understand its pathogenesis and enable identification of new therapeutic targets.

Functional magnetic resonance imaging (fMRI) is one of the rapidly developing subfields of neuroimaging research. This technology is characterized as noninvasive and straightforward in operation, is particularly well suited to clinical applications, and has wide-ranging utility for investigating the central processing of pain (Zuo & Xing, 2014). Specifically, previous studies have suggested that short periods of pain are followed by short pain-free periods, causing a rapidly changing hemodynamic response (Morton, Sandhu & Jones, 2016). Functional and structural cerebral changes have also been reported in patients with chronic pain (Davis & Moayedi, 2013). In addition, fMRI may be used to evaluate pain caused by noxious heat in healthy participants (Wager et al., 2013) and the effects of drugs on human brain activity. Furthermore, fMRI may systematically predict how analgesic drugs affect the brain (Wager & Woo, 2015). Thus, fMRI is a highly useful tool for investigating mechanisms involved in brain remodeling and enabling analgesic drug discovery.

Although there have been few relevant studies on ONFH, many researchers have investigated the cerebral mechanisms of other joint diseases such as osteoarthritis (OA) and rheumatoid arthritis (RA). RA is accompanied by changes in altered central pain processing (Jones & Derbyshire, 1997) and functional connectivity and brain structure (Schrepf et al., 2018). Chronic rheumatic pain owing to OA may be caused by central sensitivity and dysfunction of the medial pain pathway (Cottam et al., 2016). In addition, joint dysfunction was confirmed to elicit changes in brain function (Di Russo et al., 2006). In one study with a small patient cohort, abnormal patterns of brain activity were observed in ONFH patients (Feng et al., 2020). Consequently, we hypothesized that there might be changes in neural plasticity that are associated with ONFH. Moreover, complex etiology, pathogenesis, and symptoms suggest that these changes may not be limited to the sensory cortex and might occur in other brain regions.

The amplitude of low-frequency fluctuation (ALFF) and regional homogeneity (ReHo) are common methods used to describe regional features of resting-state fMRI (rs-fMRI). ALFF and ReHo define functional characteristics from different viewpoints. ALFF represents the neural activity intensity at a particular voxel by depicting the amplitude of time-series fluctuations at each voxel (Zang et al., 2007), whereas ReHo represents the importance of a specific voxel by measuring the local time-series synchronization of neighboring voxels (Zang et al., 2004). Therefore, rs-fMRI is considered a reliable and sensitive metric for studying pathological brain mechanisms (Biswal, 2012; Lee, Smyser & Shimony, 2013; Liu et al., 2013; Liu et al., 2012).

In addition to rs-fMRI, diffusion tensor imaging (DTI) is another technique that measures the direction and magnitude of water molecule diffusion within each voxel (Mori et al., 2002), and may play an important role in the pathogenesis (Guo et al., 2012a; Guo et al., 2012b). DTI has been increasingly used to investigate white matter (WM) microstructure (Van Eimeren et al., 2010). Smith et al. (2006) used tract-based spatial statistics (TBSS) to perform voxel-wise statistical comparisons of DTI data from individual participants. In the present study, probabilistic tractography was performed to enable connectivity-pattern tracking between specific regions. WM and gray matter are important components of the central nervous system, and rs-fMRI combined with DTI can comprehensively evaluate both functional and structural aspects of brain remodeling caused by a disease. This approach was successfully used to evaluate joint disease (Liu et al., 2019) and pain (Kim et al., 2013; Rogachov et al., 2018). Accordingly, we used a combination of rs-fMRI and DTI to explore functional changes in focal brain regions and structural changes in the WM of the connectome.

## Patients and Methods

### Participants

A total of 23 right-handed ONFH inpatients (14 males, nine females; aged 55.61 ± 13.79 years) who did not undergo surgery and 20 right-handed controls (12 males, eight females; aged 47.25 ± 19.35 years) were enrolled from August 2018 to August 2019. ONFH was diagnosed based on imaging and clinical examination findings by two experienced radiologists and one experienced orthopedic surgeon. Based on the Ficat classification (Sultan et al., 2019), patients with stage II and III ONFH were included. Patients were included if they were experiencing ONFH for the first time and had joint pain in the groin, buttock, or thigh areas that was described as ≥4 on a visual analog pain scale (Dworkin et al., 2005). Individuals were excluded if they had a history of cardiovascular or cerebrovascular diseases, ankylosing spondylitis, hip dysplasia, metabolic disorders, bone tumors, or psychiatric disorders. No patients received painkillers during the week before the study, and none of the control participants had any pain-related diseases. All recruited individuals had no detected brain damage (including infarcts or tumors) on conventional T1-MRI. This study was approved by the institutional review board of the Yueyang Hospital of Integrated Traditional Chinese and Western Medicine. Participants were provided written informed consent and allowed to drop out of research at any time. (No. 2018-041-01).

### MRI data acquisition

MRI data were acquired using a MAGNETOM Verio 3.0-T scanner (Siemens Healthineers, Erlangen, Germany) with a 32-channel phased array head coil. Participants were instructed to close their eyes and lie still; their heads were immobilized with foam pads, and their ears were plugged with earplugs. The rs-fMRI data were obtained using a single-pass gradient recalled echoplanar imaging (EPI) sequence with the following parameters: interleaved scanning order, slice number = 43, transverse orientation, flip angle = 90°, matrix size = 64 × 64, repetition time (TR) = 3000 ms, slice thickness = 3.0 mm, field of view (FOV) = 230 × 230 mm^2^, gap = 0 (voxel size = 3.6 × 3.6 × 3.0 mm^3^), and number of acquisitions = 200. T1-weighted magnetization-prepared rapid acquisition was performed with the following parameters: repetition time/inversion time/echo tim *e* = 1900/900/2.93 ms, flip angle = 9°, FOV = 256 × 256 mm^2^, slice thickness = 1.0 mm, sagittal acquisition, acquisition matrix = 256 × 256, and number of averages = 1. Diffusion-weighted images were acquired using a single-shot spin EPI in the axial plane: repetition time/echo time = 10000/89, flip angle 90°, slice thickness = 2.0 mm, in-plane resolution = 1.875 mm, 60 non-colinear directions (*b* = 1000 s/mm^2^), and two b0 (*b* = 0) images. T1 images of five participants (three patients and two controls) were excluded owing to poor image quality.

### Data processing

Functional images from each participant were preprocessed using the Resting-State fMRI Data Analysis Toolkit (REST; version 1.8) (Song et al., 2011), which was based on SPM12 (Wellcome Centre for Human Neuroimaging, http://www.fil.ion.ucl.ac.uk/spm) and was run through MATLAB (version 2103b; Mathworks, Natick, MA, USA). Preprocessing was performed as previously reported (Wu et al., 2019). Quality control involved bias field correction, coregistration, motion correction, and spatial normalization. Subjects were excluded according to the criterion that head motion was restricted to less than 2.5 mm of displacement or 2.5° of rotation. Regressing nuisance variables included WM, cerebrospinal fluid, and 6 head motion parameters. ALFF and ReHo were calculated for the traditional low-frequency band (0.01–0.08 Hz) (Lv et al., 2019). Spatial smoothing (Gaussian kernel of six mm full-width at half-maximum) was performed after ReHo calculation, as in previous studies (Zang et al., 2004).

Diffusion-weighted images were assessed for quality and then further processed using the FSL Diffusion Toolbox (University of Oxford Center for Functional MRI of the Brain, http://www.fmrib.ox.ac.uk/). Data processing was performed as described in our previous study (Ma et al., 2020). For a better assessment, diffusivity maps (fractional anisotropy (FA), axial diffusivity (AD), radial diffusivity (RD), and mean diffusivity (MD)) were assessed.

Fiber tracking (Behrens et al., 2007) was initiated from all voxels within the seed mask in the diffusion space to generate 5,000 streamline samples with a step length of 0.5 mm, a curvature threshold of 0.2, and a maximum of 2,000 steps. A target mask was used (right dorsolateral superior frontal gyrus), and the distribution of fiber orientations was calculated using the left dorsolateral superior frontal gyrus as a seed mask (statistically significant results in ALFF). We used FSL to identify the voxel with the maximum connectivity value within the connectivity distribution map of each participant and used thresholds to 15% of the maximum connectivity value to determine the optimum threshold value (Khalsa et al., 2014).

### Statistical analysis

SPSS (version 21.0; IBM, Armonk, NY, USA) was used to conduct our analyses in clinical data. Data are expressed as mean ± standard deviation for continuous variables and No. (%) for categorical variables. Two-sample *t*-test was used for continuous variables; and *x*^2^ test was used for categorical variables. Only clinical data findings with two-tailed *p* < 0.05 were considered significant.

The ALFF and ReHo maps were compared between the patient and control groups. Two-sample *t-* test was used for the two groups (two-tailed). AlphaSim estimation was performed for the ALFF and ReHo values using the REST toolkit. The resulting statistical map was set at *p* < 0.05 (AlphaSim correction) with a combined individual voxel *p* < 0.001. At the same time, a more strict threshold (cluster size >102 voxels) was used for each cohort to reduce the possibility of false negative results. Two-sample *t-* test was performed to assess differences between groups (two-tailed) in DTI data. The TBSS statistical method was the same as that described in our previous study (Ma et al., 2020), and the family-wise error correction was used for multiple comparisons (*p* < 0.05) (Hua et al., 2008). All numerical values were analyzed using SPSS, and statistical significance was set at *p* < 0.05.

## Results

### Demographic and clinical characteristics

Twenty-three ONFH inpatients who did not undergo surgery and 20 controls were enrolled. No significant differences in age, sex, education, past history, operation history, and diabetes were found between the two groups (Table 1).

### Functional comparisons between ONFH patients and controls

**Figure 1:**
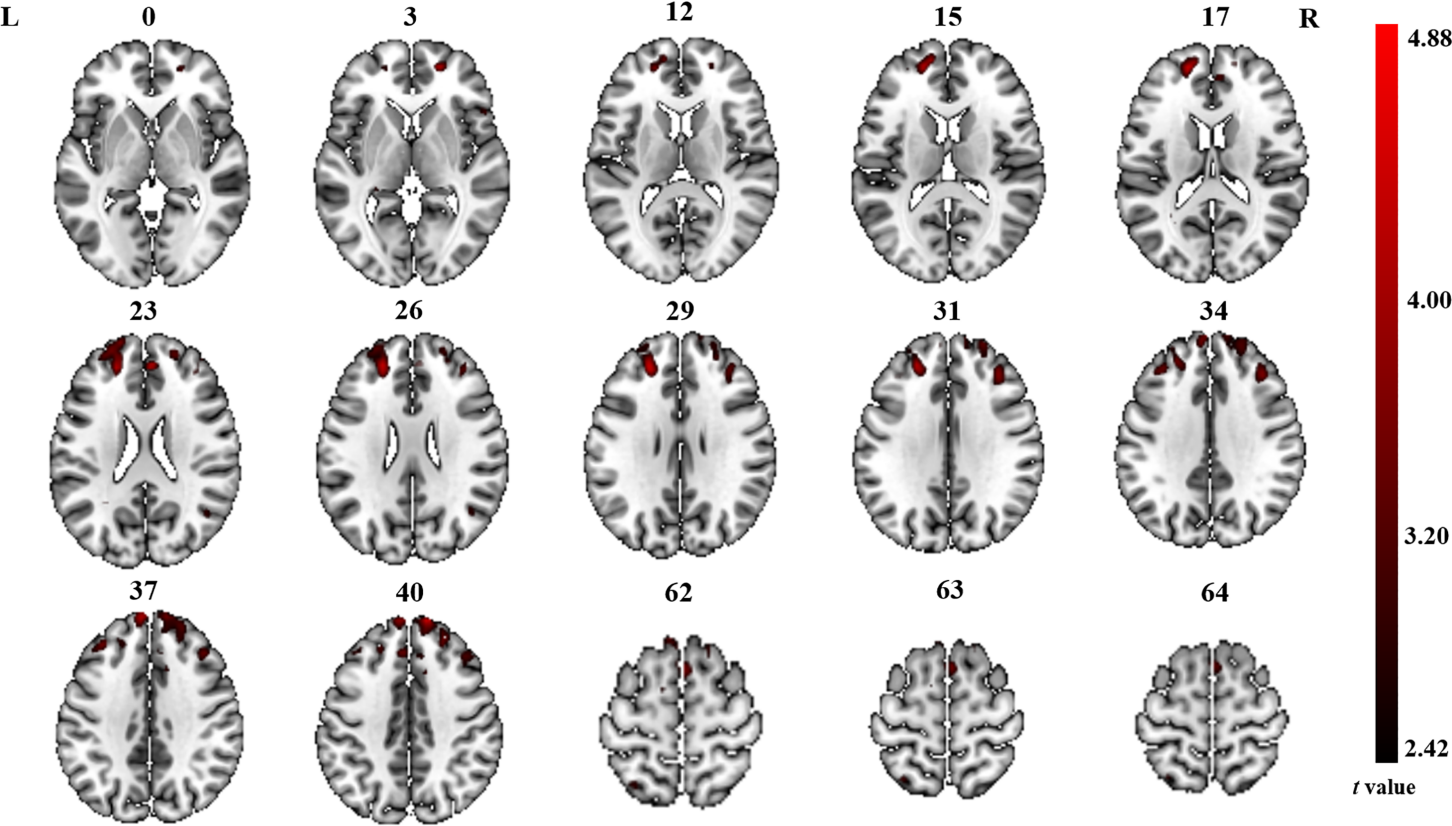
ALFF analysis. Two-sample *t*-test results are presented. Areas in red indicate significantly increased ALFF value. In the comparison of ALFF value between ONFH patients and controls, ONFH patients showed significantly increased ALFF in right medial superior frontal gyrus, left and right dorsolateral superior frontal gyrus, right middle frontal gyrus and right supplementary motor area. ALFF, low-frequency fluctuation; ONFH, osteonecrosis of the femoral head.

**Figure 2:**
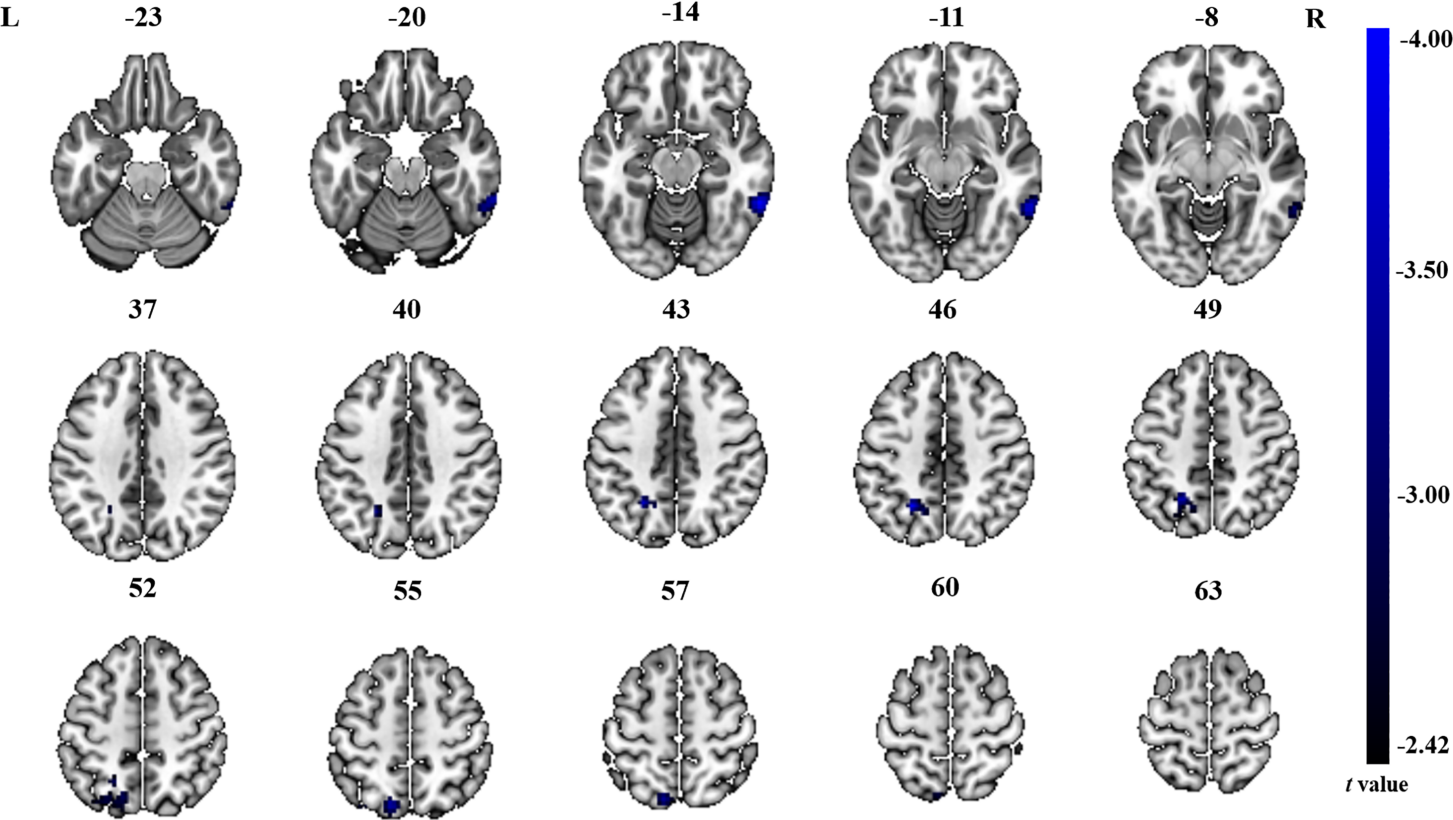
ReHo analysis. Two-sample *t*-test results are presented. Areas in blue indicate significantly decreased ReHo value. In the comparison of ReHo value between ONFH patients and controls, ONFH patients showed significantly decreased ReHo in left superior parietal gyrus and right inferior temporal gyrus. ONFH, osteonecrosis of the femoral head; ReHo, regional homogeneity.

The significant differences in ALFF and ReHo values between ONFH patients and controls are shown in Figs. 1 and 2, respectively. The comparison of ALFF values between ONFH patients and controls suggests that ONFH patients had significantly increased ALFF values in the right medial superior frontal gyrus (Brodmann area 8), left and right dorsolateral superior frontal gyrus (Brodmann area 46), right middle frontal gyrus (Brodmann area 10), and right supplementary motor area (SMA; Brodmann area 6; Fig. 1 and Table 2). The comparison of ReHo values between ONFH patients and controls suggests that ONFH patients had significantly decreased ReHo values in the left superior parietal gyrus (Brodmann area 7) and right inferior temporal gyrus (Brodmann area 20; Fig. 2 and Table 3).

**Table 1:** Demographic and clinical characteristics of the total sample.

Variables	ONFH patients (*n* = 23)	controls (*n* = 20)	*p* value
Age (y)	55.61 ± 13.79	47.25 ± 19.35	0.12^*^
Men (n,%)	14(60.9)	12(60.0)	0.95^#^
Education (years)	12.48 ± 2.84	12.30 ± 3.18	0.85^*^
Operation history(n,%)	5(21.7)	4(20)	1.00^#^
Hypertension(n,%)	2(8.7)	2(10)	1.00^#^
Diabetes(n,%)	3(13)	2(10)	1.00^#^

Notes.

* using two-sample *t*-test.

# using *x*^2^ test.

**Table 2:** Brain regions showing differences in ALFF values for patients with ONFH compared to controls.

Brain regions	Hemisphere	Cluster size	Cluster centroid MNI coordinates			*t* value
			x	y	z	
Medial superior frontal gyrus	R	267	12	57	42	4.88
Dorsolateral superior frontal gyrus	R		24	54	21	3.31
Supplementary motor area	R	244	12	12	57	3.50
Dorsolateral superior frontal gyrus	L	238	−21	45	24	4.42
Middle frontal gyrus	R	114	36	33	33	3.96

**Table 3:** Brain regions showing differences in ReHo values for patients with ONFH compared to controls.

Brain regions	Cluster size	Cluster centroid MNI coordinates			*t* value
		x	y	z	
Left superior parietal gyrus	135	−24	−57	45	−4.00
Right inferior temporal gyrus	114	60	−48	−15	−3.99

### WM microstructure comparisons between ONFH patients and controls

TBSS analysis revealed no significant differences in FA, AD, MD, or RD maps between ONFH patients and controls. When we assessed FA, AD, and MD of the WM tracts segmented by fiber-tracking DTI-MRI, we observed no significant differences between ONFH patients and controls in the left and right dorsolateral superior frontal gyrus.

## Discussion

The current multimodal study investigated resting-state brain activity and WM fiber integrity in ONFH patients. Our results indicate no significant changes in WM tract integrity in ONFH patients; however, we found abnormal brain activity in ONFH patients but not in control participants. More specifically, ONFH patients had significantly increased ALFF values in the medial superior frontal gyrus, dorsolateral superior frontal gyrus, middle frontal gyrus, and SMA and significantly decreased ReHo values in the superior parietal gyrus and inferior temporal gyrus. Our findings confirm the initial hypothesis that in ONFH patients, changes in neural plasticity are not limited to the sensory cortex and occur in several other brain regions.

Pain is a major symptom of ONFH and is usually confined to the groin area, occasionally involving the ipsilateral hip and knee or greater trochanteric area (Zhao & Hu, 2012). Enhanced activation of the prefrontal cortex (PFC), which inhibits functional connectivity between the midbrain and medial thalamus, is related to decreased pain (Morton, Sandhu & Jones, 2016). Further projections from the anterior cingulate cortex to the PFC may also be involved in the cognitive appraisal of the stimulus. Thus, the PFC has an important role in pain processing (Jones et al., 2012). We found that ONFH patients had significantly increased ALFF values in the dorsolateral superior frontal gyrus, medial superior frontal gyrus, and middle frontal gyrus. Notably, the areas that showed significantly increased ALFF values were exactly where the central portion of the PFC was located. These findings suggest that local spontaneous neuronal activity is enhanced in the PFC. Prefrontal responses to pain depend on the psychological state of the participant who may expect worsened or reduced pain (Brown & Jones, 2010). A study of arthritis patients showed that psychological characteristics and attitudes toward illness might be more important predictors of disability and pain than arthritis severity (Keefe et al., 1991). Therefore, our findings could provide therapeutic targets and crucial information on which forebrain regions are responsible for mediating adaptive responses (*e.g.*, cognitive coping strategies and attentional distraction) in ONFH patients. In addition, functional and structural changes in the PFC, including ankylosing spondylitis (Liu et al., 2020), irritable bowel syndrome (Mao et al., 2020), chronic lower back pain (Yu et al., 2020), and neuropathic pain (Li et al., 2020), have been demonstrated in other pain studies. In a recent rs-fMRI study (Feng et al., 2020), researchers revealed an abnormal pattern of brain activity in ONFH patients and found that the most significant increase in ALFF occurred in pain-related brain regions, which is consistent with our results.

In our current study, we found significantly increased ALFF values in the SMA (forepart) of the brain. The function of the SMA is related to the planning of movements, whereas that of the pre-SMA is related to the learning of new movement sequences. Neural activity in the pre-SMA is higher when individuals perform a newer motor sequence; once the motor sequence is learned, the neural activity in the pre-SMA is reduced. The SMA of the cerebral cortex is also considered a part of the pain matrix, which integrates sense and body movements (Hanakawa, 2012; Iadarola et al., 1998). Notably, limitations in motion are another chief complaint of osteonecrosis. In ONFH, patients must change their original activity habits owing to pain and limitation of movement; thus, local spontaneous neuronal activity is enhanced in the SMA. Horiuchi et al. also found that structural changes in the SMA are associated with chronic myelopathic pain (Horiuchi et al., 2020).

In contrast to ALFF, areas of altered ReHo were observed only in the superior parietal and inferior temporal gyri in our current study. The superior parietal gyrus belongs to the secondary sensory cortex and is involved in sensory information integration, analysis, and spatial localization (Galletti et al., 1997). The superior parietal gyrus is connected to the primary sensory motor cortex by round-trip fibers, which are responsible for the high-level integration function. The final comprehensive analysis and adjudication are conducted in each brain area to enable the transmission of information. Notably, considering the ALFF results, we found that the brain regions showing significant differences (between ONFH patients and controls) were likely part of the sensorimotor network (SMN). The SMN includes somatosensory and motor regions and extends to the SMA (Chenji et al., 2016). Previous studies have demonstrated that many conditions, including stroke (Chen et al., 2019), Parkinson’s disease (Li et al., 2019), bipolar disorder (Martino et al., 2020), and irritable bowel syndrome (Labus et al., 2019), can cause dysfunction of the SMN. Although no studies on ONFH have been published to date, Zhang et al. (2018) and Sevel et al. (2020) detected changes in the SMN in cases of lower limb amputation and repeated delayed onset muscle soreness.

Our results showed decreased ReHo values in the inferior temporal gyrus in ONFH patients, which is closely related to cognitive function. Multi-item working memory refers to an individual’s ability to temporarily store and operate information when performing cognitive tasks, an important cognitive function that is frequently used in everyday life. Working memory, which can monitor, process, and maintain information, is considered the core foundation of advanced human cognitive activities. More importantly, the inferior temporal gyrus can reflect higher-level information processing (Costers et al., 2020). The decreased ReHo values in the inferior temporal gyrus observed in our current study may represent a change in higher-level information processing prompted by changes in the living conditions of ONFH patients.

Despite the significantly abnormal brain functions in ONFH patients compared with controls in our present study, DTI was not significantly different between the two groups. This result may indicate that no WM microstructural changes occurred in ONFH patients at stage II and III, which may be because the TBSS analysis was strictly corrected for multiple comparisons. Although the probability of false-positive results was avoided, the probability of false-negative results increased, and different brain regions might be submerged by multiple comparison corrections. If WM microstructural differences did occur in those patients, it should be confirmed by increasing the number of staged cases in further research.

This was a preliminary pioneer-pilot study of brain mechanisms in ONFH patients compared with those in healthy controls. This study had several limitations. First, the sample size was relatively small and may have limited the statistical power. Consequently, the study results must be interpreted cautiously. Second, it may be more precise to compare patients with different types and stages of ONFH. Third, participants were instructed to close their eyes and lie still during the imaging; however, no measures were taken to ensure that the participants were not asleep.

## Conclusions

Our current findings revealed possible changes in brain functional plasticity in ONFH patients, with resulting functional impairments potentially occurring in different brain regions. Despite its limitations, this study broadens our understanding of the neural mechanisms involved in ONFH and may provide an explanation for refractory pain associated with the disease.

## Supplemental Information

10.7717/peerj.11759/supp-1 Supplemental Information 1 Raw data exported from ALFF analysis between case group and control group

10.7717/peerj.11759/supp-2 Supplemental Information 2 Raw data exported from ReHo analysis between case group and control group

10.7717/peerj.11759/supp-3 Supplemental Information 3 Clinical data

10.7717/peerj.11759/supp-4 Supplemental Information 4 Raw data exported from fractional anisotropy [FA], axial diffusivity [AD], radial diffusivity [RD], and mean diffusivity [MD] between case group and control group after fiber tracking was finished

10.7717/peerj.11759/supp-5 Supplemental Information 5 Codebook

## Additional Information and Declarations

### Data Availability

The following information was supplied regarding data availability: The raw measurements are available in the Supplemental Files.

## References

[ref-1] Behrens TE, Berg HJ, Jbabdi S, Rushworth MF, Woolrich MW (2007). Probabilistic diffusion tractography with multiple fibre orientations: what can we gain?. NeuroImage.

[ref-2] Biswal BB (2012). Resting state fMRI: a personal history. NeuroImage.

[ref-3] Brown CA, Jones AK (2010). Meditation experience predicts less negative appraisal of pain: electrophysiological evidence for the involvement of anticipatory neural responses. Pain.

[ref-4] Chen H, Shi M, Zhang H, Zhang YD, Geng W, Jiang L, Wang Z, Chen YC, Yin X (2019). Different patterns of functional connectivity alterations within the default-mode network and sensorimotor network in basal ganglia and pontine stroke. Medical Science Monitor.

[ref-5] Chenji S, Jha S, Lee D, Brown M, Seres P, Mah D, Kalra S (2016). Investigating default mode and sensorimotor network connectivity in amyotrophic lateral sclerosis. PLOS ONE.

[ref-6] Costers L, Van Schependom J, Laton J, Baijot J, Sjøgård M, Wens V, De Tiège X, Goldman S, D’Haeseleer M, D’Hooghe MB, Woolrich M, Nagels G (2020). Spatiotemporal and spectral dynamics of multi-item working memory as revealed by the n-back task using MEG. Human Brain Mapping.

[ref-7] Cottam WJ, Condon L, Alshuft H, Reckziegel D, Auer DP (2016). Associations of limbic-affective brain activity and severity of ongoing chronic arthritis pain are explained by trait anxiety. NeuroImage: Clinical.

[ref-8] Davis KD, Moayedi M (2013). Central mechanisms of pain revealed through functional and structural MRI. Journal of Neuroimmune Pharmacology.

[ref-9] Di Russo F, Committeri G, Pitzalis S, Spitoni G, Piccardi L, Galati G, Catagni M, Nico D, Guariglia C, Pizzamiglio L (2006). Cortical plasticity following surgical extension of lower limbs. NeuroImage.

[ref-10] Dworkin RH, Turk DC, Farrar JT, Haythornthwaite JA, Jensen MP, Katz NP, Kerns RD, Stucki G, Allen RR, Bellamy N, Carr DB, Chandler J, Cowan P, Dionne R, Galer BS, Hertz S, Jadad AR, Kramer LD, Manning DC, Martin S, McCormick CG, McDermott MP, McGrath P, Quessy S, Rappaport BA, Robbins W, Robinson JP, Rothman M, Royal MA, Simon L, Stauffer JW, Stein W, Tollett J, Wernicke J, Witter J (2005). Core outcome measures for chronic pain clinical trials: IMMPACT recommendations. Pain.

[ref-11] Feng S, Li B, Li G, Hua X, Zhu B, Li X, Lu W, Xu J (2020). Abnormal spatial patterns of intrinsic brain activity in osteonecrosis of the femoral head: a resting-state functional magnetic resonance imaging study. Frontiers in Human Neuroscience.

[ref-12] Galletti C, Fattori P, Kutz DF, Battaglini PP (1997). Arm movement-related neurons in the visual area V6A of the macaque superior parietal lobule. European Journal of Neuroscience.

[ref-13] Guo W, Liu F, Liu Z, Gao K, Xiao C, Chen H, Zhao J (2012a). Right lateralized white matter abnormalities in first-episode, drug-naive paranoid schizophrenia. Neuroscience Letters.

[ref-14] Guo WB, Liu F, Xue ZM, Gao K, Wu RR, Ma CQ, Liu ZN, Xiao CQ, Chen HF, Zhao JP (2012b). Altered white matter integrity in young adults with first-episode, treatment-naive, and treatment-responsive depression. Neuroscience Letters.

[ref-15] Hanakawa T (2012). Neural mechanisms underlying deafferentation pain: a hypothesis from a neuroimaging perspective. Journal of Orthopaedic Science.

[ref-16] Hopkins C, Genant HK (2020). Editorial for Guidelines for clinical diagnosis and treatment of osteonecrosis of the femoral head in adults (2019 version). Journal of Orthopaedic Translation.

[ref-17] Horiuchi Y, Tsuji O, Komaki Y, Fujiyoshi K, Hikishima K, Konomi T, Nagoshi N, Watanabe K, Matsumoto M, Horiuchi K, Nakamura M (2020). Characteristic cerebral structural changes identified using voxel-based morphometry in patients with post-surgical chronic myelopathic pain. Spinal Cord.

[ref-18] Hua K, Zhang J, Wakana S, Jiang H, Li X, Reich DS, Calabresi PA, Pekar JJ, Van Zijl PC, Mori S (2008). Tract probability maps in stereotaxic spaces: analyses of white matter anatomy and tract-specific quantification. NeuroImage.

[ref-19] Hungerford DS (2002). Osteonecrosis: avoiding total hip arthroplasty. Journal of Artroplasty.

[ref-20] Iadarola MJ, Berman KF, Zeffiro TA, Byas-Smith MG, Gracely RH, Max MB, Bennett GJ (1998). Neural activation during acute capsaicin-evoked pain and allodynia assessed with PET. Brain.

[ref-21] Jones AK, Derbyshire SW (1997). Reduced cortical responses to noxious heat in patients with rheumatoid arthritis. Annals of the Rheumatic Diseases.

[ref-22] Jones AK, Huneke NT, Lloyd DM, Brown CA, Watson A (2012). Role of functional brain imaging in understanding rheumatic pain. Current Rheumatology Reports.

[ref-23] Keefe FJ, Caldwell DS, Martinez S, Nunley J, Beckham J, Williams DA (1991). Analyzing pain in rheumatoid arthritis patients. Pain coping strategies in patients who have had knee replacement surgery. Pain.

[ref-24] Khalsa S, Mayhew SD, Chechlacz M, Bagary M, Bagshaw AP (2014). The structural and functional connectivity of the posterior cingulate cortex: comparison between deterministic and probabilistic tractography for the investigation of structure-function relationships. NeuroImage.

[ref-25] Kim JY, Kim SH, Seo J, Kim SH, Han SW, Nam EJ, Kim SK, Lee HJ, Lee SJ, Kim YT, Chang Y (2013). Increased power spectral density in resting-state pain-related brain networks in fibromyalgia. Pain.

[ref-26] Labus JS, Osadchiy V, Hsiao EY, Tap J, Derrien M, Gupta A, Tillisch K, Le Nevé B, Grinsvall C, Ljungberg M, Öhman L, Törnblom H, Simren M, Mayer EA (2019). Evidence for an association of gut microbial Clostridia with brain functional connectivity and gastrointestinal sensorimotor function in patients with irritable bowel syndrome, based on tripartite network analysis. Microbiome.

[ref-27] Lee MH, Smyser CD, Shimony JS (2013). Resting-state fMRI: a review of methods and clinical applications. American Journal of Neuroradiology.

[ref-28] Li Z, Chen J, Lin Y, Zhou M, Cai Q, Li X, Wu Z, Chen X, Yang X, Zhu X, Lu J, Zhang L, Liu B, Luo X, Xu P (2019). Reduced regional activity and functional connectivity within sensorimotor network in Parkinson’s patients with restless legs syndrome. Molecular Pain.

[ref-29] Li X, Xu LS, Xu YF, Yang Q, Fang ZX, Yao M, Chen WY (2020). The gene regulatory network in different brain regions of neuropathic pain mouse models. European Review for Medical and Pharmacological Sciences.

[ref-30] Liu F, Guo W, Liu L, Long Z, Ma C, Xue Z, Wang Y, Li J, Hu M, Zhang J, Du H, Zeng L, Liu Z, Wooderson SC, Tan C, Zhao J, Chen H (2013). Abnormal amplitude low-frequency oscillations in medication-naive, first-episode patients with major depressive disorder: a resting-state fMRI study. Journal of Affective Disorders.

[ref-31] Liu J, Chen L, Tu Y, Chen X, Hu K, Tu Y, Lin M, Xie G, Chen S, Huang J, Liu W, Wu J, Xiao T, Wilson G, Lang C, Park J, Tao J, Kong J (2019). Different exercise modalities relieve pain syndrome in patients with knee osteoarthritis and modulate the dorsolateral prefrontal cortex: a multiple mode MRI study. Brain, Behavior, and Immunity.

[ref-32] Liu F, Hu M, Wang S, Guo W, Zhao J, Li J, Xun G, Long Z, Zhang J, Wang Y, Zeng L, Gao Q, Wooderson SC, Chen J, Chen H (2012). Abnormal regional spontaneous neural activity in first-episode, treatment-naive patients with late-life depression: a resting-state fMRI study. Progress in Neuro-Psychopharmacology and Biological Psychiatry.

[ref-33] Liu Q, Liao Z, Zhang Y, Lin C, He B, Fang L, Tu L, Zhao M, Wu X, Gu J (2020). Pain- and Fatigue-related functional and structural changes in ankylosing spondylitis: an fRMI study. Frontiers in Medicine.

[ref-34] Lv Y, Li L, Song Y, Han Y, Zhou C, Zhou D, Zhang F, Xue Q, Liu J, Zhao L, Zhang C, Han X (2019). The local brain abnormalities in patients with transient ischemic attack: a resting-state fMRI study. Frontiers in Neuroscience.

[ref-35] Ma J, Hua XY, Zheng MX, Wu JJ, Huo BB, Xing XX, Ding W, Xu JG (2020). Structural remodeling secondary to functional remodeling in advanced-stage peripheral facial neuritis. Neurological Science.

[ref-36] Mao CP, Chen FR, Huo JH, Zhang L, Zhang GR, Zhang B, Zhou XQ (2020). Altered resting-state functional connectivity and effective connectivity of the habenula in irritable bowel syndrome: a cross-sectional and machine learning study. Human Brain Mapping.

[ref-37] Martino M, Magioncalda P, Conio B, Capobianco L, Russo D, Adavastro G, Tumati S, Tan Z, Lee HC, Lane TJ, Amore M, Inglese M, Northoff G (2020). Abnormal functional relationship of sensorimotor network with neurotransmitter-related nuclei via subcortical-cortical loops in manic and depressive phases of bipolar disorder. Schizophrenia Bulletin.

[ref-38] May A (2008). Chronic pain may change the structure of the brain. Pain.

[ref-39] Mori S, Frederiksen K, Van Zijl PC, Stieltjes B, Kraut MA, Solaiyappan M, Pomper MG (2002). Brain white matter anatomy of tumor patients evaluated with diffusion tensor imaging. Annals of Neurology.

[ref-40] Morton DL, Sandhu JS, Jones AK (2016). Brain imaging of pain: state of the art. Journal of Pain Research.

[ref-41] Rogachov A, Cheng JC, Hemington KS, Bosma RL, Kim JA, Osborne NR, Inman RD, Davis KD (2018). Abnormal low-frequency oscillations reflect trait-like pain ratings in chronic pain patients revealed through a machine learning approach. Journal of Neuroscience.

[ref-42] Schrepf A, Kaplan CM, Ichesco E, Larkin T, Harte SE, Harris RE, Murray AD, Waiter GD, Clauw DJ, Basu N (2018). A multi-modal MRI study of the central response to inflammation in rheumatoid arthritis. Nature Communications.

[ref-43] Sevel L, Boissoneault J, Alappattu M, Bishop M, Robinson M (2020). Training endogenous pain modulation: a preliminary investigation of neural adaptation following repeated exposure to clinically-relevant pain. Brain Imaging and Behavior.

[ref-44] Smith SM, Jenkinson M, Johansen-Berg H, Rueckert D, Nichols TE, Mackay CE, Watkins KE, Ciccarelli O, Cader MZ, Matthews PM, Behrens TE (2006). Tract-based spatial statistics: voxelwise analysis of multi-subject diffusion data. NeuroImage.

[ref-45] Song XW, Dong ZY, Long XY, Li SF, Zuo XN, Zhu CZ, He Y, Yan CG, Zang YF (2011). REST: a toolkit for resting-state functional magnetic resonance imaging data processing. PLOS ONE.

[ref-46] Sultan AA, Mohamed N, Samuel LT, Chughtai M, Sodhi N, Krebs VE, Stearns KL, Molloy RM, Mont MA (2019). Classification systems of hip osteonecrosis: an updated review. International Orthopaedics.

[ref-47] Van Eimeren L, Grabner RH, Koschutnig K, Reishofer G, Ebner F, Ansari D (2010). Structure-function relationships underlying calculation: a combined diffusion tensor imaging and fMRI study. NeuroImage.

[ref-48] Wager TD, Atlas LY, Lindquist MA, Roy M, Woo CW, Kross E (2013). An fMRI-based neurologic signature of physical pain. New England Journal of Medicine.

[ref-49] Wager TD, Woo CW (2015). fMRI in analgesic drug discovery. Science Translational Medicine.

[ref-50] Wu JJ, Lu YC, Zheng MX, Hua XY, Xu JG, Ding W, Shan CL (2019). Motor control deficits in facial synkinesis patients: neuroimaging evidences of cerebral cortex involvement. Neural Plasticity.

[ref-51] Yu S, Li W, Shen W, Edwards RR, Gollub RL, Wilson G, Park J, Ortiz A, Cao J, Gerber J, Mawla I, Chan ST, Lee J, Wasan AD, Napadow V, Kaptchuk TJ, Rosen B, Kong J (2020). Impaired mesocorticolimbic connectivity underlies increased pain sensitivity in chronic low back pain. NeuroImage.

[ref-52] Zang YF, He Y, Zhu CZ, Cao QJ, Sui MQ, Liang M, Tian LX, Jiang TZ, Wang YF (2007). Altered baseline brain activity in children with ADHD revealed by resting-state functional MRI. Brain and Development.

[ref-53] Zang Y, Jiang T, Lu Y, He Y, Tian L (2004). Regional homogeneity approach to fMRI data analysis. NeuroImage.

[ref-54] Zhang J, Zhang Y, Wang L, Sang L, Li L, Li P, Yin X, Qiu M (2018). Brain functional connectivity plasticity within and beyond the sensorimotor network in lower-limb amputees. Frontiers in Human Neuroscience.

[ref-55] Zhao DW, Hu YC (2012). Chinese experts’ consensus on the diagnosis and treatment of osteonecrosis of the femoral head in adults. Orthopaedic Surgery.

[ref-56] Zuo XN, Xing XX (2014). Test-retest reliabilities of resting-state FMRI measurements in human brain functional connectomics: a systems neuroscience perspective. Neuroscience and Biobehavioral Reviews.

