# Supplementary material for: Alterations in brain structure and function in patients with osteonecrosis of the femoral head: a multimodal MRI study
Source: PeerJ. 2021 Aug 20;9:e11759. doi: 10.7717/peerj.11759 (PMC8381875; doi:10.7717/peerj.11759)
Supplement: Supplemental Information 5 [file peerj-09-11759-s005.docx]

group

1. Control group
2. Case group

Gender

1. Male
2. Female

Operation

1. No
2. Yes

Hypertension

1. No
2. Yes

DM

1. No
2. Yes
